# The complete mitochondrial genome of the springtail *Allacma fusca*, the internal phylogenetic relationships and gene order of Symphypleona

**DOI:** 10.1080/23802359.2020.1800425

**Published:** 2020-07-30

**Authors:** Francesco Nardi, Claudio Cucini, Chiara Leo, Francesco Frati, Pietro Paolo Fanciulli, Antonio Carapelli

**Affiliations:** Department of Life Sciences, University of Siena, Siena, Italy

**Keywords:** Symphypleona, Sminthuridae, springtails mitogenomics, Collembola

## Abstract

Symphypleona (*sensu stricto*) are a group of Collembola (=springtails) that, despite displaying some variation in gene order, have been poorly investigated under the phylomitogenomic perspective. How families and subfamilies of this taxon are evolutionary related is still partially unknown. For this reason we sequenced, and herein described, the complete mitochondrial genome sequence of *Allacma fusca* (Sminthuridae). This sequence, alongside others from the literature, is here used to study the phylogenetic relationships among Symphypleona.

Among Collembola, the order Symphypleona (*sensu stricto*) accounts for over 1200 species (https://www.collembola.org/). Traditionally, this taxon was grouped with Neelipleona because of the shared spherical body shape in Symphypleona *sensu lato*. Despite this similar morphological feature, recent molecular evidence established that Symphypleona *s.s.* are more closely related to springtails characterized by an elongated body (Entomobryomorpha and Poduromorpha) than to Neelipleona, supporting this latter as the basal group of springtails (Leo et al. 2019a, 2019b; Sun et al. 2020). However, knowledge on the systematic position of families and subfamilies within the order is still limited due, among other issues, to the restricted number of mitogenomes available. *Allacma fusca*, a member of family Sminthuridae, is a holarctic species and is strictly confined to humid soils (Fanciulli et al. 2009). In this study, we focus on the evolutionary relationships within Symphypleona *s.s.*, at family and subfamily level, describing and comparing the mitogenome of *A. fusca* with those of Symphypleona available on GenBank.

A pool of five individuals of *A. fusca* (voucher specimen ID: AFU_05, preserved at Life Sciences Department of the University of Siena), collected in the Feniglia dunes (Grosseto, Italy; 42°24′42′′N 11°12′43′′E) in November 2019, was used for the analysis. Total DNA was extracted using the QIAmp^®^ UCP DNA kit and sequenced on a HISeq 2500 platform (Illumina, San Diego, CA) at Macrogen Europe. Resulting reads were assembled using two *de novo* software: MEGAHIT version 1.2.9 ( Li et al. 2015) on trimmed sequences and NOVOPlasty version 3.8.3 on untrimmed sequences (Dierckxsens et al. 2017). Single base ambiguities (resulting from the use of a pool of individuals) were resolved on a majority rules basis by remapping reads on interested regions using bbmap version 38.84 (https://www.sourceforge.net/projects/bbmap/) and visualizing alignments in IGV version 2.8.2 ( Robinson et al. 2011). The final mitochondrial DNA (mtDNA) sequence was annotated as described in Leo et al. (2019a). *Allacma fusca* protein-coding genes (PCGs) were aligned with homologous sequences from available Symphypleona complete mitogenomes plus two outgroups (Figure 1) as in Leo et al. (2019c). The resulting data matrix was portioned in 39 subsets (one for each of the three codon positions of the 13 PCGs) and analyzed with PartitionFinder 2.1.1 (Lanfear et al. 2016). The selected models (HKY + Γ, HKY + I+Γ, GTR + I, and GTR + I+Γ) and partitioning scheme were used in MrBayes version 3.2 (Ronquist et al. 2012), through the CIPRES Science Gateway (Miller et al. 2010), for a Bayesian phylogenetic analysis using four chains for 10^6^ generations with a of burn-in of 0.25 and a sampling frequency of one tree every 1000 iterations.

**Figure 1.**
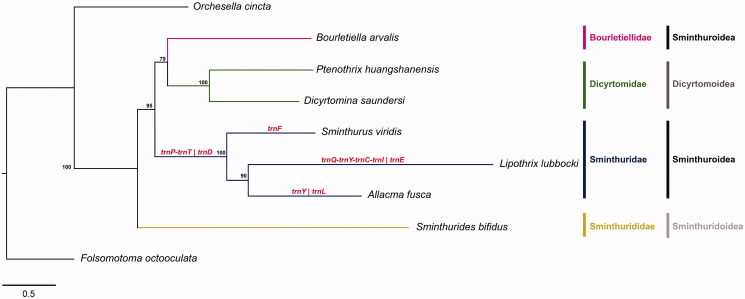
Bayesian phylogenetic tree inferred based on the 13 mitochondrial protein-coding genes sequences of *Folsomotoma octooculata* (NC024155, outgroup), *Orchesella cincta* (NC032283, outgroup), *Sminthurides bifidus* (MK423964), *Bourletiella arvalis* (NC039558), *Ptenotrix huangshanensis* (MK423965), *Dicyrtomina saundersi* (NC044134), *Sminthurus viridis* (NC010536), *Lipothrix lubbocki* (MK431899) and *Allacma fusca* (MT547779). Gene order translocations in Sminthuridae are shown in red. Posterior probability is shown at nodes.

The complete mtDNA of *A. fusca* is a circular molecule of 15,111 bp in length. Overall, a bias in the nucleotide composition is observed toward As (39.4%) and Ts (32.6%), with respect to Cs (16.4%) and Gs (11.6%). It encodes for the set of 37 genes (13 PCGs, 22 tRNAs, and 2 rRNAs) commonly observed in metazoan mitochondrial genomes. Gene arrangement differs from both the ancestral pancrustacean gene order and from alternative gene orders previously described in springtails (Boore et al. 1998; Carapelli et al. 2007; Leo et al. 2019a; Sun et al. 2020). *Allacma fusca* shares with the other two representatives of family Sminthuridae*, Lipotrix lubbocki* and *Sminthurus viridis, two* translocations (*trnD* and exchange between *trnT* and *trnP*) that may be tentatively regarded as synapomorphies for the family. Moreover, at variance with *L. lubbocki* and *S. viridis*, *A. fusca* displays two additional translocations (*trnY* and *trnL1)* that appear autapomorphic. In particular, the *trnY* translocation occurs from *nad2-trnW-trnC-trnY-cox1* (ancestral pancrustacean GO) to *nad2-trnY-trnW-trnC-cox1*; the *trnL1* translocation occurs from *nad1-trnL1-rrnL-trnV-rrnS* (ancestral pancrustacean GO) to *nad1-rrnL-trnV-rrnS-trnL1* (Figure 1). Our analysis confirms that the ancestral pancrustacean GO model is plesiomorphic for the order Symphypleona *s.s.*,but changed in the Sminthuridae family. The phylogenetic analysis, performed using aligned concatenated PCG sequences, produced the tree depicted in Figure 1. All nodes display high support with the exception of the one joining Bourletiellidae with Dicyrtomidae. The tree indicates that: (a) the Sminthurididae (represented by *Sminthurides bifidus*) are basal to all the other Symphypleona; (b) Dicyrtomidae and Sminthuridae (here represented by more than one species) are monophyletic groups; (c) the Sminthuroidea superfamily may be paraphyletic, because of the placement of *Bourletiella arvalis* (Bourletiellidae) as the sister group to the Dicyrtomidae clade. Nevertheless, this latter result receives only modest supported and should be reevaluated based on an enlarged taxonomic coverage of Bourletiellidae.

## Data Availability

The data that support the findings of this study are openly available in GenBank of NCBI with reference number MT547779 at https://www.ncbi.nlm.nih.gov/nuccore/MT547779.
